# Sleep-Disordered Breathing in Patients with Heart Failure: New Trends in Therapy

**DOI:** 10.1155/2013/459613

**Published:** 2013-07-28

**Authors:** Anna Kazimierczak, Paweł Krzesiński, Krystian Krzyżanowski, Grzegorz Gielerak

**Affiliations:** Department of Cardiology and Internal Diseases, Military Institute of Medicine, Szaserow Street 128, 04-141 Warsaw, Poland

## Abstract

Heart failure (HF) is a growing health problem which paradoxically results from the advances in the treatment of etiologically related diseases (especially coronary artery disease). HF is commonly accompanied by sleep-disordered breathing (SDB), which may directly exacerbate the clinical manifestations of cardiovascular disease and confers a poorer prognosis. Obstructive sleep apnoea predominates in mild forms while central sleep apnoea in more severe forms of heart failure. Identification of SDB in patients with HF is important, as its effective treatment may result in notable clinical benefits to the patients. Continuous positive airway pressure (CPAP) is the gold standard in the management of SDB. The treatments for central breathing disorders include CPAP, bilevel positive airway pressure (BPAP), and adaptive servoventilation (ASV), with the latter being the most modern method of treatment for the Cheyne-Stokes respiration and involving ventilation support with a variable synchronisation dependent on changes in airflow through the respiratory tract and on the patient's respiratory rate. ASV exerts the most favourable effect on long-term prognosis. In this paper, we review the current state of knowledge on the diagnosis and treatment of SDB with a particular emphasis on the latest methods of treatment.

## 1. Introduction

Heart failure (HF) is a condition in which the heart—as a result of functional abnormalities—is unable to generate an appropriate blood flow to meet the body's metabolic needs. The rapid development of effective treatments for cardiovascular disease, which is the most common cause of hospitalisation and mortality in Poland, has contributed to the significant growth of the population of HF patients. The prevalence of HF in Poland and worldwide has now risen to epidemic proportions. There is therefore an ongoing search for new treatments that would improve the prognosis and quality of life in patients with HF. One should be aware that the therapeutic success is not only dependent on the treatment of cardiovascular disorders but also on the treatment of comorbid conditions that affect the function of the kidneys and liver and of the respiratory, nervous, and endocrine systems.

There has been a growing focus in recent years on sleep-disordered breathing (SDB), a previously underestimated issue in the population of patients with HF [1]. Heart failure patients may suffer from both obstructive and central breathing disorders. In milder forms of HF (NYHA functional classes I and II), obstructive sleep apnoea (OSA) is more common, while central sleep apnoea (CSA) is the typical form of breathing disorders in patients with more severe HF (NYHA functional classes III and IV) [2]. In patients with severe HF, CSA takes the form of Cheyne-Stokes respiration (CSR). All these abnormalities are significantly associated with the pathophysiology of HF, which is why methods of noninvasive ventilation are becoming increasingly popular with cardiologists. It turns out that the number of patients that are eligible for this treatment and could significantly benefit from it is rising. The aim of this paper is to review the diagnostic and therapeutic options available for HF patients with SDB.

## 2. Heart Failure: A Clinical Challenge

The estimated prevalence of HF in the European population is 0.4–2.0%, which translates into about 10 million patients being affected with this condition in Europe. In a Polish study of outpatients of over 65 years of age, HF was diagnosed in 53%. The incidence increases with age and is higher in men than in women. The mean age of patients with HF is about 74 years. Coronary artery disease (mainly myocardial infarction) and hypertension are currently believed to be the most common causes of HF, although it may also be secondary to arrhythmias, valvular heart disease, and pericardial diseases [3, 4].

Another problem in patients with HF is the poor prognosis, especially in cases in which causal treatment is not possible. Nearly 50% of the patients die within 4 years of diagnosis with over half of the patients with severe HF dying within a year. In chronic advanced HF the mean 5-year survival rate is estimated at 25–50% in men and 38–50% in women and is comparable to that observed in cancer. Poor remote prognosis is also associated with asymptomatic myocardial dysfunction [3, 5, 6].

The last few years have seen considerable progress in the management of HF in terms of pharmacotherapy, electrotherapy, and cardiac surgery. HF is a complex problem, and its management requires combining several strategies each affecting a different pathophysiological mechanism. Contemporary cardiology not only offers treatment of the signs and symptoms but also addresses the causes and consequences of cardiac dysfunction in a multidirectional manner. Conventional treatment is currently complemented by revascularisation procedures, valvular surgery, cardiomyoplasty, cardiac resynchronisation therapy, implantable cardioverter-defibrillators, radiofrequency ablation, heart transplantation, ventricular assist devices (including the so-called artificial heart), laser treatment, cell therapy, and gene therapy [7]. 

 Despite the expansion of methods that support its management, HF continues to pose a great challenge to cardiologists, as each of the known methods of treatment is associated with a number of limitations for the individual patient. These limitations are the driving force in the search for new treatment strategies for HF.

## 3. Heart Failure and Sleep-Disordered Breathing

**Figure 1:**
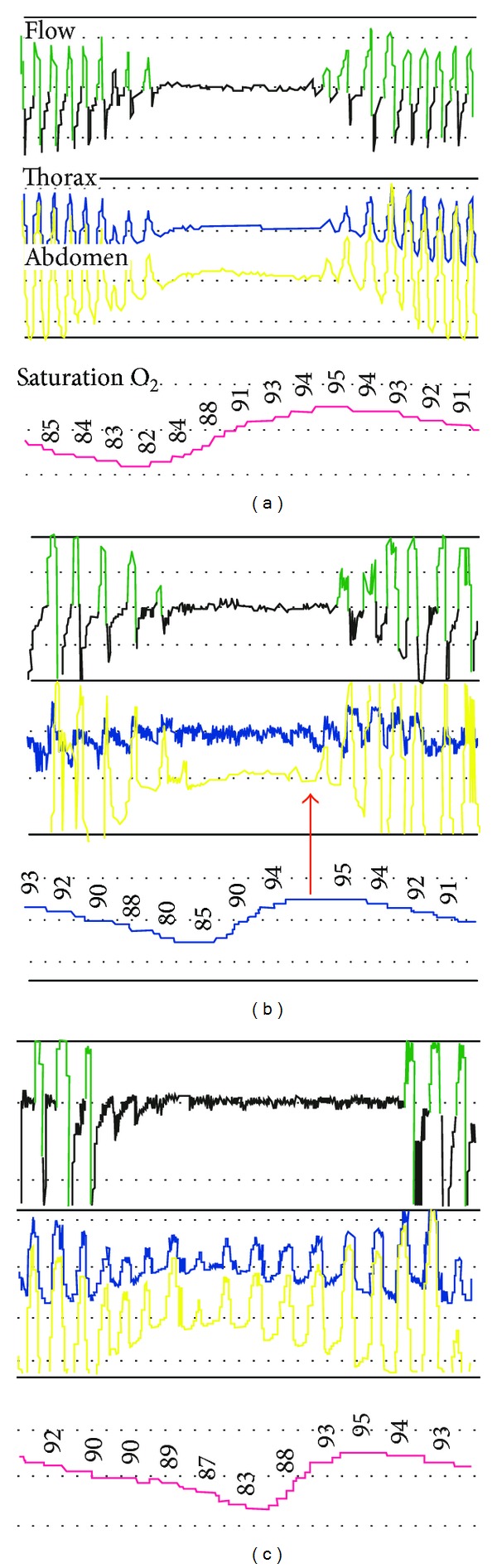
Polysomnographic recordings of central sleep apnoea (a), mixed sleep apnoea (b), and obstructive sleep apnoea (c). Episodes of apnoea paralleled by the absence of respiratory movements of the chest (blue line) and abdomen (yellow line) indicate a central nature of the breathing disorder. The crescendo-decrescendo breathing pattern typical of Cheyne-Stokes respiration is evident (a). Episodes of apnoea with the initial absence of the respiratory movements of the chest and abdomen, followed by their resumption (red arrow), indicate a mixed-type breathing disorder (b). Episodes of apnoea paralleled by the respiratory movements of the chest and abdomen confirm the obstructive nature of the breathing disorder (c).

SDB in HF may take the following forms:

- *obstructive sleep apnoea* (OSA), defined as repeated episodes of limitation or cessation of airflow through the respiratory tract during sleep as a result of upper airway collapse,
- *central sleep apnoea* (CSA), associated with the lack of impulses from the respiratory centre and, secondarily, with the cessation of respiratory movements of the chest and abdomen that result in the cessation of airflow through the respiratory tract. Cheyne-Stokes respiration (CSR) is a special form of this disorder,
- *mixed sleep apnoea* (MSA), in which there is an initial cessation of breathing of central origin with a collapse of the soft parts of the pharynx, followed by a resumption of function of the respiratory centre, while the upper airway is collapsed (Figure 1).

Data on the incidence of SDB in the population of patients with HF are equivocal. In a study by Oldenburg et al. [8], in the group of 700 patients with HF, 252 (36%) presented with OSA and 280 (40%) with CSA. These findings are consistent with the results of a study by Schulz et al. conducted in 2007 [9] in 203 patients with EF below 40% (NYHA functional classes II and III), in which patients with OSA accounted for 43% and those with CSA for 28% of the patients. According to older studies, about 40% of patients with CHF had CSA and about 11% had OSA [1]. According to Lipkin [10], about 60% of patients with CHF are affected by SDB with 36% of the patients being affected by CSA, 12% by OSA, and the rest by a combination of both types of SDB [10].

## 4. Consequences of Sleep-Disordered Breathing

**Figure 2:**
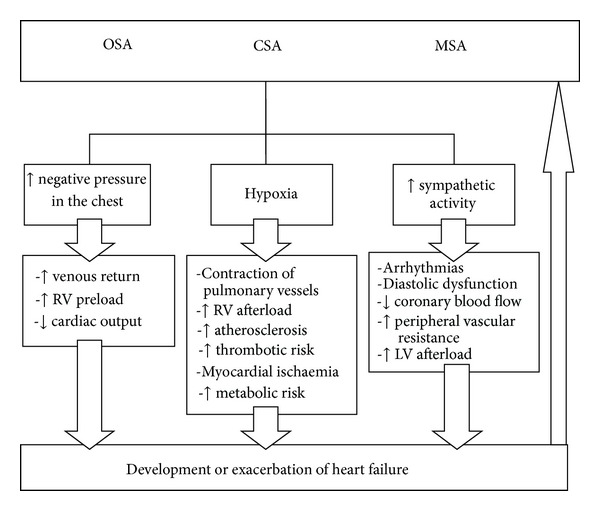
Consequences of sleep-disordered breathing. CSA: central sleep apnoea, LV: left ventricle, MSA: mixed sleep apnoea, OSA: obstructive sleep apnoea, and RV: right ventricle.

Multiple pathophysiological mechanisms underlying SDB have been documented in the literature (Figure 2). SDB has been shown to lead to excessive activation of the sympathetic system and to increased blood catecholamine levels, increased blood pressure, increased peripheral vascular resistance, increased pulmonary artery pressure, increased right and left ventricular afterload, episodic decreases in oxygen saturation, and abnormal control of baroreceptor reflexes [11–14]. 

Especially overnight rostral fluid shift influences the severity of both OSA and CSA. This effect is mainly seen in men with HF. Fluid displacement to the neck may facilitate OSA, mainly because of pharyngeal obstruction. Higher resistance of the airways results in decreased in volume of ventilation and increased PaCO_2_.

On the other hand, increased fluid accumulation in the lungs can induce reflex ventilation. As a result, PaCO_2_ decreases below the apnoea threshold and the number of CSA episodes increases [15–17]. In HF patients with dominant OSA, Yumino et al. [18] observed a strong negative correlation between the overnight change in leg fluid volume and the change in neck circumference (*r* = −0.780,   *P* < 0.001) and AHI (*r* = −0.881,   *P* < 0.001) but no association with PaCO_2_. However, in CSA dominant patients, the overnight reduction in leg fluid volume was related to an increase in AHI (*r* = −0.919,   *P* < 0.001), neck circumference (*r* = −0.568,   *P* = 0.013), and lower PaCO_2_  (*r* = 0.569,   *P* = 0.009). The association between rostral fluid shift and SDB was also observed in patients with end-stage chronic disease. In this group, the reduction in leg fluid volume correlated with apnoea-hypopnea time (*P* = 0.001) [19]. 

SDB leads to fluctuations in heart rate (bradycardia during the apnoeic pauses with subsequent tachycardia upon awakening) and to cardiac arrhythmias and conduction disorders.

The apnoea-related changes in heart rate occur in the setting of considerable fluctuations in chest pressure, hypoxia, and acid-base disorders. Bradyarrhythmias are caused by the activation of the vagus nerve during the apnoea, and the tachycardia at the end of the apnoea is caused by the increased levels of catecholamines. SDB may be a pathophysiological contributing factor for atrial fibrillation as a result of the autonomic dysfunction and the elevated atrial pressure and atrial enlargement caused by the apnoea. Severe apnoea is associated with a fourfold higher risk atrial fibrillation and more than a threefold higher risk of nonsustained ventricular tachycardia [20]. In a study by Kanagala et al. [21], coexistence of untreated OSA in patients undergoing a successful electrical cardioversion for atrial fibrillation increased the likelihood of the recurrence of this arrhythmia, which positively correlated with the severity of OSA. Effective treatment with CPAP significantly decreased the risk of recurrence of atrial fibrillation, a very important risk factor for stroke. It has also been demonstrated that SDB in patients with low ejection fraction considerably increases the risk of serious ventricular arrhythmias and that most of cardiac deaths among patients with SDB occur during the night [22, 23].

SDB also leads to increased risk of thrombosis, vascular wall injury, and increased atherogenic processes [24]. It also interferes with hormone secretion, increases the risk of metabolic abnormalities, and leads to excessive daytime sleepiness [25]. As a result, the cardiovascular system during sleep does not undergo the physiological relaxation but continues to function under conditions of excessive stimulation. 

SDB coexisting with HF is the direct cause of exacerbation of the signs and symptoms of the latter, which is why it is justified to claim that treatment of the former allows to suppress the unfavourable pathophysiological mechanisms and may play a significant role in improving the clinical condition and prognosis in patients with HF.

## 5. Obstructive Sleep Apnoea

The problem of OSA is most commonly associated with secondary hypertension. It should, however, be borne in mind that long-lasting untreated OSA may also lead to HF as a result of the chronically excessive pre- and postloading of the myocardium resulting from systemic hypertension. OSA in hypertension is increasingly diagnosed in Poland, which is evidenced by the increasing number of units dealing with the diagnosis of SDB at specialist centres for the management of hypertension. The prognostic significance of OSA has been confirmed in the Sleep Heart Health Study, identifying this breathing disorder as an independent risk factor for HF [2].

The gold standard in the management of obstructive breathing disorders is continuous positive airway pressure (CPAP). The use of prosthetic devices allows to reduce the number of apnoeas and improve the efficacy of antihypertensive treatment but also to increase left ventricular ejection fraction (LVEF), improve systolic function, and reduce left ventricular end-systolic dimension, left ventricular afterload, and arrhythmias [26–29].

## 6. Central Sleep Apnoea and Cheyne-Stokes Respiration

According to the American Academy of Sleep Medicine (2007) [30], Cheyne-Stokes respiration is defined as an abnormal pattern of breathing characterised by at least 3 consecutive cycles of cyclical crescendo and decrescendo change in breathing amplitude and at least 1 of the following:

1. five or more central apnoeas or hypopneas per hour of sleep ((apnoea/hypopnea index) AHI central ≥5),
2. the cyclic crescendo and decrescendo change in breathing amplitude has a duration of at least 10 consecutive minutes.

**Figure 3:**
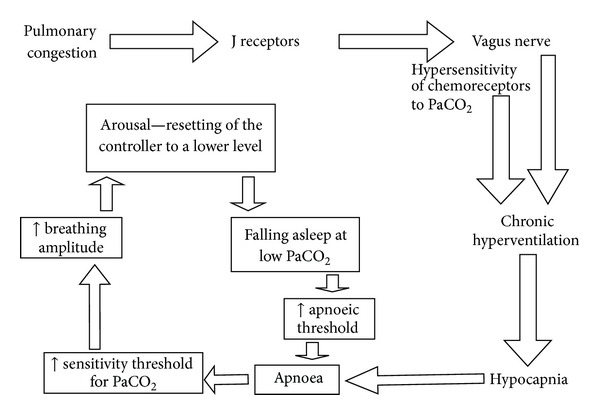
Pathomechanism of Cheyne-Stokes respiration.

The signs of CSA are similar to those of OSA (excessive daytime sleepiness, frequent arousals during sleep with choking, morning fatigue and headaches, and complaints of sleeplessness, Table 1). These may be partially masked by the manifestations of HF. The classic crescendo-decrescendo pattern of breathing is most typical of HF [31] and is closely associated with circulatory disturbances (Figure 3). Stimulation of the J mechanoreceptors by pulmonary congestion through the vagus nerve stimulates the respiratory centre. Increased work of the respiratory muscles results in chronic hyperventilation and moderate hypocapnia. This mechanism is further exacerbated by the increased sympathetic activity, which is responsible for the increased sensitivity of chemoreceptors to carbon dioxide partial pressure (PaCO_2_). During sleep, the threshold value of PaCO_2_ increases and results in apnoea. In this setting, even a moderate hypocapnia leads to the disappearance of signals from the respiratory centre and leads to central apnoea. The gradual increase in PaCO_2_ eventually reaches the sensitivity threshold and restores normal breathing function. The amplitude of airflow gradually increases, reaches the maximum in the hyperventilation phase, and then gradually decreases until apnoea ensues. The patient wakes up and the apnoea threshold decreases until the patient falls asleep again. The same low level of PaCO_2_ that does not cause apnoea when the patient is awake may lead to apnoea when the patient is asleep. This cycle of hyperventilation and apnoeas is supported by the increased sensitivity of chemoreceptors to PaCO_2_, oscillations of PaCO_2_ below and above the apnoea threshold, and repeated episodes of arousal [32]. The increased duration of flow in HF and the secondary delay of the cerebral chemical regulation of breathing relative to PaCO_2_ changes and partial pressure of oxygen in the blood (PaO_2_) are jointly responsible for the crescendo-decrescendo pattern of the respiratory amplitude.

There is a close correlation between the severity of HF and that of CSR. At the same time, CSR accelerates the progression of HF, decreases the time to heart transplantation, and increases the risk of death 2- to 3-fold [33]. Studies have demonstrated a considerable prognostic value of CSR in patients with HF, comparable to that of clinical and echocardiographic data [33]. In severe HF, CSR—although it is most typical of night-time sleep—may still extend to daytime hours.

In fact, CSR extending to daytime hours is a common clinical problem. In a study by Brack et al. [34], CSR was present in 62% of HF patients at night and in 16% of HF patients during the day. CSR occupying more than 10% of daytime activity has proved to be an independent predictor of mortality. The development of exertional oscillatory ventilation (EOV) has also been shown to be a strong factor conferring poor prognosis, as evidence of advanced instability of the breathing pattern control. It is suggested that the presence of EOV is an even stronger negative predictor than CSR that is present only during sleep [35, 36].

The coexistence of OSA and CSA in patients with HF has its pathophysiological background. Tkacova et al. [37] observed that over the course of a single night, OSA predominates at the beginning and CSA predominates at the end of the night. The results revealed that the shift from OSA to CSA is associated with a reduction in PaCO_2_. The potential mechanism involved in the overnight increase in CSA events is: worsening hypoxia, increasing ventilatory responsiveness to CO_2_, and worsening of pulmonary congestion caused by deterioration in cardiac function. Increased sympathetic nervous activity and blood pressure caused by OSA contribute to the impaired systolic and diastolic function of heart, decrease in cardiac output, and increase in left ventricular filling pressure and pulmonary wedge pressure. The proportion of both types of sleep apnoea can also vary within an individual over longer periods of time. According to another study by Tkacova et al. [38], the shift between the two types can occur in both directions. Changes from predominantly OSA to CSA are associated with reductions in CO_2_ and longer periodic breathing cycle, while changes from CSA to OSA are associated with the opposite phenomena (increase in PtcCO_2_ and shortening of the periodic breathing cycle). Moreover, the variations in breathing disorders are related to deterioration of cardiac function. 

## 7. Treatment of Sleep-Disordered Breathing

The treatment of SDB in patients with HF has been shown to increase the time to heart transplantation and improve NYHA functional class [31]. In patients with HF, the mainstay of treatment of CSA involves the treatment of the underlying illness, as it may prevent SDB.

Undoubtedly, correct pharmacological treatment of HF decreases the severity of CSR. Nevertheless, attempts at using nonstandard pharmacotherapy in the management of CSR have failed. Theophylline, a stimulant of the respiratory centre that increases its sensitivity to hypercapnia, has been considered a potentially beneficial agent, although the results of the relevant studies are not convincing. A study by Javaheri et al. [39] showed that several days of treatment with theophylline merely led to a decrease in central apnoeas and desaturation episodes in patients with stable HF but did not affect the ejection fraction. The use of theophylline is further limited by its adverse effects, mainly cardiac arrhythmias. Acetazolamide, on the other hand, induces metabolic acidosis, which stimulates ventilation. It has been shown to decrease the number of central apnoeas without, however, improving haemodynamic parameters or the quality of sleep [40]. In addition, acetazolamide may also cause hypokalaemia, which exerts a proarrhythmic effect. These two agents have therefore failed to gain acceptance for the treatment of breathing disorders in HF, and researchers have focused on other methods as potential treatments.

Attempts have also been made to assess the impact of electrotherapy on breathing disorders in patients with HF, expecting an improvement associated with an overall beneficial clinical effect in this group of patients. Cardiac resynchronisation therapy has proved the most effective, as it reduces CRS by improving the pumping function of the heart, as confirmed in a recently published meta-analysis [41]. One study showed a beneficial effect of atrial pacing at a rate 15 beats faster than the mean nocturnal heart rate resulting in a reduction in the number of both central and obstructive apnoeas [42], although subsequent studies failed to confirm these correlations. CSR may also be improved by cardiac surgery, such as implantation of an artificial mitral valve [43] or heart transplantation [1].

Some of the other potential treatment modalities for CSA dedicated to HF patients, also in long-term treatment, include passive oxygen therapy, inspiratory positive airway pressure (CPAP, bilevel positive airway pressure (BPAP)) and adaptive servoventilation (ASV).

### 7.1. Oxygen Therapy

Oxygen therapy affects chemoreceptors and decreases their sensitivity to PaCO_2_, leading to an increased hyperventilation response to hypercapnia that results from apnoea. Nocturnal oxygen therapy increases PaCO_2_ above threshold of the respiratory centre sensitivity, reduces the severity of central apnoeas, and suppresses sympathetic activation in patients with HF and CSR [44]. While oxygen therapy has been confirmed to decrease serum levels of brain natriuretic peptide [45], it has not been shown to improve LVEF [46] or significantly change the quality of sleep and cognitive function [47]. Oxygen therapy for CSA may slightly reduce AHI but does not improve sleep structure [48]. Transformation of CSA into obstructive and mixed apnoeas has also been observed as a result of oxygen therapy [49]. Oxygen therapy is therefore a method that does not provide satisfactory clinical benefits.

### 7.2. Continuous Positive Airway Pressure (CPAP)

The use of CPAP in patients with HF and CSR improves cardiac function and manifestations of HF [50], improves ejection fraction and decreases sympathetic activation [51, 52], reduces relative mortality risk, and decreases the number of heart transplantations [53]. By reducing intrathoracic and transmural pressures during systole and diastole, CPAP reduces left ventricular afterload, and by reducing venous return and left and right ventricular end-diastolic volumes, it also decreases preload [54].

Not all the patients with central breathing disorders respond to CPAP in terms of resolution of apnoea. In a study by Javaheri, the beneficial effect in the form of CSR improvement was observed in a mere 43% of the patients [55]. CPAP has proved more beneficial in dilated cardiomyopathy than in HF secondary to ischaemic heart disease (a more pronounced reduction in right ventricular end-diastolic and end-systolic volumes than in left ventricular end-diastolic and end-systolic volumes) [56]. According to some authors, the efficacy of oxygen therapy and CPAP in reducing AHI in patients with HF and CSR is similar [57]. Unfortunately, CPAP provided for CSR to patients with HF does not increase survival. In the prospective CANPAP study, Bradley et al. showed a similar percentage of patients who died or required heart transplantation in the group of patients receiving CPAP and in the control group. There were also no differences in the quality of life or the number of hospitalisations, although an improvement in ejection fraction and a decrease in sympathetic activity were observed [58]. Arzt et al. conducted a repeat analysis of the same study and divided the CPAP group into patients responding with an AHI reduction below 15/h and those in whom no such response had been seen. The analysis showed that an early significant reduction in AHI after initiation of CPAP in patients with HF translates into an improved survival until transplantation [59]. Buckle et al. [60] investigated the effect of using CPAP for one night and did not observe any effects on CSA in patients with HF. The results obtained by Davies et al. [61], who used CPAP for 2 weeks, were similar. What is more, the use of CPAP may be harmful, as it decreases intrathoracic pressure, end-diastolic volume and blood pressure in some of the patients with HF (especially in those with atrial fibrillation) [62].

### 7.3. Bilevel Positive Airway Pressure (BPAP)

BPAP is intended for patients who do not tolerate CPAP well. Willson et al. [63] observed a reduction in AHI and arousal index after initiation of BPAP, although this was not paralleled by a significant improvement in sleep structure, that is, by increases in NREM phases 3 and 4 and in REM. However, G. Johnson and C. Johnson [64] compared the outcomes of using CPAP and BPAP in various breathing disorders during sleep and showed the greatest intensity of central apnoeas in the case of BPAP.

### 7.4. Adaptive Servoventilation (ASV)

The most modern treatments for CSR include ventilation support devices with variable synchronisation dependent on flow and breathing rate. We therefore present this form treatment modality in the greatest detail here.

ASV machines track minute ventilation or peak flow and adjust support pressure to stabilise ventilation or the mean amplitude of peak airflow during respiration. The algorithm used by these machines also assesses the direction, magnitude, and frequency of momentary changes in airflow and compares these with the target values. Based on a series of measurements of airflow for each respiration ventilation support is adjusted, which ensures its synchronisation with the patient's respiratory effort. If the patient hypoventilates, the machine increases the support pressure, and in response to hyperventilation, it rapidly lowers it. After the breathing pattern has normalised, the machine provides minimal support pressure in order to avoid excessive ventilation and hypocapnia. If an apnoea occurs, the machine switches to time mode with breathing rate support.

There are reports suggesting that the greatest benefits of using ASV to normalise breathing in CSA are found in patients with CSA secondary to HF.

Pepperell et al. used ASV for 4 weeks in 30 patients and observed a decrease in AHI, neurohormonal activity, N-terminal prohormone of brain natriuretic peptide (NT-proBNP) levels, and excessive daytime sleepiness in patients with HF and CSR [65]. A reduction in AHI, arousal index, and excessive daytime sleepiness and an improvement in the quality of life with ASV have been confirmed in another, slightly longer study [66]. Shädlich et al., in a study investigating 12-month treatment with ASV in 20 patients, showed improved sleep structure (increase in slow-wave sleep without changes in REM sleep), improved cardiac function (as assessed by ejection fraction), and increased 6-minute walk test distance [67]. In another study, using ASV for one night led to an increase in PaO_2_ and SaO_2_, a decrease in NT-proBNP levels, and a decrease in serum catecholamine levels [68]. Treatment with ASV for 2 weeks, versus oxygen therapy, resulted in a significant increase in left ventricular ejection fraction, improvement in physical exercise capacity, increase in slow-wave sleep, increase in arterial blood saturation, and other changes [69]. Philippe et al. conducted a randomised study in 25 patients to compare the results of using ASV versus CPAP for 6 months and showed a complete resolution of CSR only in the ASV group [70]. In this group, statistically significant improvements in LVEF and the quality of life were also observed as well as a better compliance at 6 months of treatment (compliance at 3 months was comparable in both groups). Also, in a study by Morgenthaler et al., the efficacy in reducing AHI and arousal index in patients with CSR was greater with ASV than with CPAP [71].

Fietze et al. compared the outcomes of ASV versus BPAP used for 6 weeks in 30 patients with LVEF values below 45% and CSA and showed a significant reduction in AHI and increase in LVEF with both treatment modalities with a more pronounced decrease in AHI observed in the ASV group and a more pronounced increase in LVEF in the BPAP group [72]. A study by Kasai et al. to assess various treatment modalities (ASV versus BPAP versus CPAP) used for one night showed that ASV was most effective in improving sleep structure by prolonging the duration of slow-wave sleep [73].

The comparison of the efficacy of various modalities of treatment for breathing disorders in a study by Teschler et al. [48], after one night at a random order: passive oxygen therapy, CPAP, BPAP, and ASV, showed ASV to be the most effective in reducing apnoeas and hypopneas, in reducing arousal index, and in improving the quality of sleep. Beneficial changes in sleep structure were only observed in the cases of BPAP and ASV and a comparison of these two modalities revealed a statistically significant prolongation of NREM and REM in the ASV group. The greatest reduction in AHI was observed in the ASV (by 86%, a statistically significant difference) compared to the other treatment modalities (oxygen therapy, CPAP, and BPAP, where the AHI reductions were 37%, 40%, and 67%, resp.,). Oldenburg et al. showed an improvement in cardiopulmonary exercise testing parameters (increased peak VO_2_ and VAT) and echocardiographic parameters (increased LVEF) and a decrease in NT-proBNP levels as a result of several months of treatment with ASV [74].

Results of a large (1260 patients) randomised study, SERVE-HF, are soon to be published. The study is aimed at comparing the effects of ASV versus optimum pharmacotherapy on long-term prognosis in patients with HF and central breathing disorders.

### 7.5. Phrenic Nerve Stimulation in the Treatment of CSA

Phrenic nerve stimulation is the most recently developed (although still investigational) method of invasive treatment for CSA in patients with HF. The phrenic nerve is stimulated by electrodes of an implantable device similar to implantable cardiac pacemakers. Preliminary observations made in a pilot study in 16 patients using an external stimulator fitted for 48 hours showed a 90% reduction in CSA paralleled by a decrease in total AHI of 48% [75]. Further studies are ongoing to assess the outcomes and safety of long-term phrenic nerve stimulation for the treatment of CSA in patients with HF.

## 8. Limitations of Noninvasive Ventilation Support Devices

Treatment with ventilation support devices as a form of noninvasive treatment is associated with relatively few side effects (dry mouth, nose and throat, conjunctival irritation, exacerbation of hypotension in patients with low baseline blood pressure). These are not life threatening and are easily manageable. Subjective intolerability of these devices seems to be the greatest problem stemming from the discomfort associated with using an external device and from the necessity to operate it correctly. The cost, especially in the case of ASV, is also an important limitation for many patients.

## 9. Perspectives

Studies conducted in recent years provide an increasing body of evidence to support the relationship between SDB and cardiovascular disease. A development of the currently used technologies is to be expected with a particular focus on improving the comfort of using the device by the patient. The significance of treatment of comorbidities, including the risk factors for rostral fluid displacement, is also bound to increase.

Studies at the level of metabolic pathways, neuroendocrine regulation, and chronic inflammatory activation are also gaining importance. In recent years, particular attention has been paid to the relationship of OSA and glucose metabolism, whose disorders are a component of metabolic syndrome. OSA has been recognised as an independent risk factor for insulin resistance and diabetes mellitus [76, 77], and an obesity-independent effect of OSA on lipid abnormalities has also been suggested [78]. Further studies are required to determine whether defining OSA as a “stand-alone” cardiovascular risk factor independent of visceral obesity is correct.

Studies conducted in recent years also indicate that inflammation—as evidenced by the inflammatory infiltrate in the upper airways and the increased levels of proinflammatory cytokines (IL-1beta, IL-2, and TNF-alpha)—plays an important role in the pathogenesis of OSA [79, 80]. The relationship between SDB and inflammation will undoubtedly be a topic of future research, which might result in new treatments.

The significance of CSR in patients with HF also requires a more in-depth investigation. The ongoing studies might determine whether treatment of SDB improves long-term prognosis in this group. The question arises whether CSR is a marker of the severity of HF or a significant comorbidity that affects the prognosis. The significance of SDB in HF determines the new directions in the management of these patients.

Studies to investigate the relationship between SDB and cardiovascular disease are some of the best examples of how big a role can the interdisciplinary approach to the patient play in the development of medicine.

## 10. Conclusion

Drawing attention to SDB that coexists with HF and to its appropriate treatment using noninvasive ventilation support devices may prove a safe and effective form that supports the treatment of the underlying illness. It may be a valuable complement to the management of the growing population of patients with HF. By reducing the incidence of acute exacerbations of HF and of the number of hospitalisations and by improving the functional status of patients with HF, treatment of SDB may be an important element that limits the costs of overall care of these patients. This, however, requires further studies that will identify the optimal forms of treatment for SDB, which will establish indications for its use and identify patients that are most likely to benefit clinically.

## Figures and Tables

**Table 1:** Clinical manifestations of sleep-disordered breathing.

Night-time manifestations	Daytime manifestations
(i) Snoring (OSA) (ii) Apnoeas (iii) Dyspnoea (iv) Excessive sweating (v) Nocturia (vi) Palpitations (vii) Sleep fragmentation (viii) Dry mouth	(i) Excessive daytime sleepiness (ii) Attention disorders (iii) Morning headache (iv) Sexual dysfunction (v) Emotional disorders

## List of Abbreviations

AHI: Apnoea/hypopnea index
ASV: Adaptive servoventilation
BPAP: Bilevel positive airway pressure
CPAP: Continuous positive airway pressure
CSA: Central sleep apnoea
CSR: Cheyne-Stokes respiration
EOV: Exertional oscillatory ventilation
HF: Heart failure
LVEF: Increase left ventricular ejection fraction
MSA: Mixed sleep apnoea
NYHA: New York Heart Association
NT-proBNP: N-terminal prohormone of brain natriuretic peptide
NREM: Nonrapid eye movement (phase)
OSA: Obstructive sleep apnoe
PaCO_2_: Carbon dioxide partial pressure
PaO_2_: Oxygen partial pressure
REM: Rapid Eye Movement (phase)
SDB: Sleep-disordered breathing.
